# MicroRNA Signatures and Machine Learning Models for Predicting Cardiotoxicity in HER2-Positive Breast Cancer Patients

**DOI:** 10.3390/ph18121908

**Published:** 2025-12-18

**Authors:** Maria Anastasiou, Evangelos Oikonomou, Panagiotis Theofilis, Maria Gazouli, George-Angelos Papamikroulis, Athina Goliopoulou, Vasiliki Tsigkou, Vasiliki Skandami, Angeliki Margoni, Kyriaki Cholidou, Amanda Psyrri, Konstantinos Tsioufis, Flora Zagouri, Gerasimos Siasos, Dimitris Tousoulis

**Affiliations:** 1 Section of Medical Oncology, 2nd Propaedeutic Department of Internal Medicine and Clinical Research Institute, Attikon University Hospital, National and Kapodistrian University of Athens Medical School, 11527 Athens, Greece; miriamanastasiou9@gmail.com (M.A.); psyrri237@yahoo.com (A.P.); 2 3rd Department of Cardiology, Sotiria Chest Disease Hospital, National and Kapodistrian University of Athens Medical School, 11527 Athens, Greece; boikono@gmail.com (E.O.); gapapa02@gmail.com (G.-A.P.); agoliopoulou@gmail.com (A.G.); bikytsigkoy@yahoo.gr (V.T.); ger_sias@hotmail.com (G.S.); 3 1st Cardiology Department, “Hippokration” Athens General Hospital, National and Kapodistrian University of Athens Medical School, 11527 Athens, Greece; panos.theofilis@hotmail.com (P.T.); ktsioufis@gmail.com (K.T.); 4 Laboratory of Biology, Department of Basic Medical Sciences, National and Kapodistrian University of Athens Medical School, 11527 Athens, Greece; maria.gazouli@gmail.com; 5 Department of Microbiology, “Hippokration” Athens General Hospital, 11527 Athens, Greece; 6 Department of Biological Chemistry, National and Kapodistrian University of Athens Medical School, 11527 Athens, Greece; angeliki.margoni@gmail.com; 7 1st Respiratory Medicine Department, Sotiria Chest Disease Hospital, National and Kapodistrian University of Athens Medical School, 11527 Athens, Greece; kg.cholidou@yahoo.gr; 8 Department of Clinical Therapeutics, Alexandra Hospital, National and Kapodistrian University of Athens Medical School, 11528 Athens, Greece; florazagouri@yahoo.co.uk

**Keywords:** cardiotoxicity, breast cancer, machine learning, microRNA, biomarker

## Abstract

**Background:** HER2-positive breast cancer patients receiving chemotherapy and targeted therapy (including anthracyclines and trastuzumab) face an elevated risk of cardiotoxicity, which can lead to long-term cardiovascular complications. Identifying predictive biomarkers is essential for early intervention. Circulating microRNAs (miRNAs), known regulators of gene expression and cardiovascular function, have emerged as potential indicators of cardiotoxicity. This study aims to evaluate the differential expression of circulating miRNAs in HER2-positive breast cancer patients undergoing chemotherapy and to assess their prognostic ability for therapy-induced cardiotoxicity using machine learning models. **Methods:** Forty-seven patients were assessed for cardiac toxicity at baseline and every 3 months, up to 15 months. Blood samples were collected at baseline. MiRNA expression profiling for 84 microRNAs was performed using the miRCURY LNA miRNA PCR Panel. Differential expression was calculated via the 2^−∆∆Ct^ method. The five most upregulated and five most downregulated miRNAs were further assessed using univariate logistic regression and receiver operating characteristic (ROC) analysis. Five machine learning models (Decision Tree, Random Forest (RF), Support Vector Machine (SVM), Gradient Boosting Machine (GBM), k-Nearest Neighbors (KNN)) were developed to classify cardiotoxicity based on miRNA expression. **Results:** Forty-five miRNAs showed significant differential expression between cardiac toxic and non-toxic groups. ROC analysis identified hsa-miR-155-5p (AUC 0.76, *p* = 0.006) and hsa-miR-124-3p (AUC 0.75, *p* = 0.007) as the strongest predictors. kNN, SVM, and RF models demonstrated high prognostic accuracy. The decision tree model identified hsa-miR-17-5p and hsa-miR-185-5p as key classifiers. SVM and RF highlighted additional miRNAs associated with cardiotoxicity (SVM: hsa-miR-143-3p, hsa-miR-133b, hsa-miR-145-5p, hsa-miR-185-5p, hsa-miR-199a-5p, RF: hsa-miR-185-5p, hsa-miR-145-5p, hsa-miR-17-5p, hsa-miR-144-3p, and hsa-miR-133a-3p). Performance metrics revealed that SVM, kNN, and RF models outperformed the decision tree in overall prognostic accuracy. Pathway enrichment analysis of top-ranked miRNAs demonstrated significant involvement in apoptosis, p53, MAPK, and focal adhesion pathways, all known to be implicated in chemotherapy-induced cardiac stress and remodeling. **Conclusions:** Circulating miRNAs show promise as biomarkers for predicting cardiotoxicity in breast cancer patients. Machine learning approaches may enhance miRNA-based risk stratification, enabling personalized monitoring and early cardioprotective interventions.

## 1. Introduction

Anthracyclines and trastuzumab are two of the basic antineoplastic regimens that are used as (neo)adjuvant treatment for human epidermal growth factor receptor 2 (HER2)-positive early breast cancer. Despite their efficacy, they are associated with cardiotoxicity, which can lead to severe cardiac dysfunction and heart failure [1,2]. As the combination of these agents leads to a 27% incidence of cardiotoxicity [3], their sequential (two-step) administration is the standard of care for the last two decades, which lowers the levels of cardiotoxicity at 3–4% [4,5,6]. Another strategy in favor of reducing the anthracycline-induced cardiotoxicity is to limit the cumulative dose of anthracyclines. Guidelines recommend a cumulative dose of doxorubicin between 400 and 550 mg/m^2^ to minimize the risk of heart failure [7]. Lastly, the most challenging action to place a limit on cardiotoxicity is the early detection and management in order to prevent long-term cardiac dysfunction and improve patients’ quality of life.

Prognostic biomarkers play a pivotal role in identifying patients at risk of developing cardiotoxicity from antineoplastic treatment. Troponins, especially high-sensitivity troponin, and N-terminal pro b-type natriuretic peptide (NT-proBNP) have been investigated as diagnostic and prognostic tool for detection of cardiotoxicity, but the results are inconsistent. Some studies failed to associate both biomarkers with cardiotoxicity [8,9,10], while others confirmed the hypothesis of correlation for at least one of the biomarkers [11,12,13,14]. Novel plasma biomarkers such as myeloperoxidase, soluble ST2, growth differentiation factor 15 and miRNAs are under investigation for their contribution in prediction and detection of cardiotoxicity. Despite this considerable progress, there is a persistent lack of validated biomarkers for early detection of cardiotoxicity, which substantially limits proactive clinical management and individual risk stratification. This knowledge gap is the basis of our study’s hypothesis and underscores the rationale for leveraging machine learning models in order to address the previously mentioned unmet need.

Therefore, we evaluate miRNAs as prognostic biomarkers of antineoplastic treatment-induced cardiotoxicity in patients with breast cancer treated with epirubicin plus cyclophosphamide, followed by taxane and trastuzumab.

## 2. Results

### 2.1. Differential MiRNA Expression

**Figure 1:**
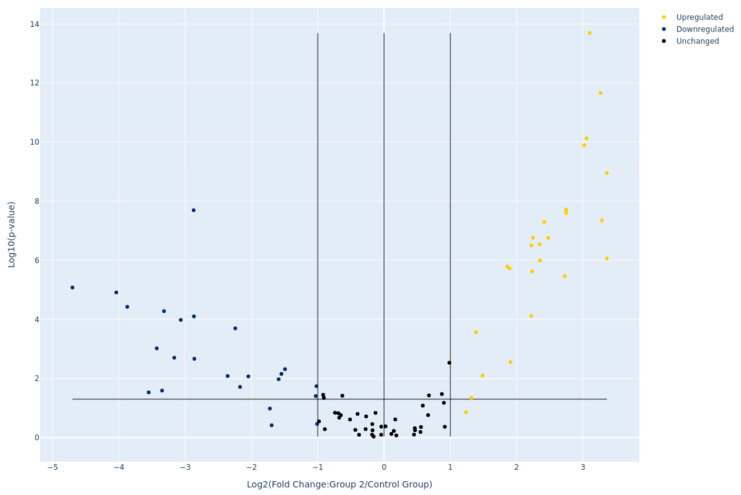
**Volcano plot of all 84 miRNAs in our assay.** Red dots represent miRNAs with a −1 <log_2_FC> 1 (fold regulation of at least 2) and −log_10_*p* > 1.31 (*p* < 0.05, Student *t*-test).

**Figure 2:**
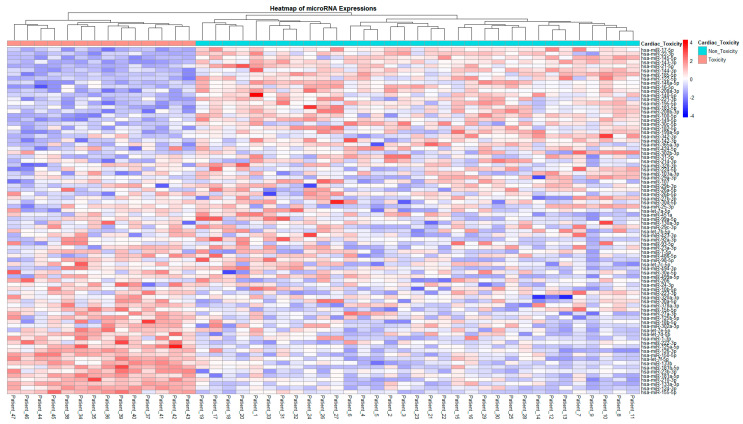
**Heatmap of scaled miRNA Expressions values across patients with and without cardiac toxicity.** Each column corresponds to a patient, and each row represents a miRNA. The heatmap colors reflect standardized expression levels (blue = downregulated, red = upregulated). Patients are ordered by cardiac toxicity status (left: toxicity, right: non-toxicity), and miRNAs are sorted by effect size (mean difference between groups) and hierarchically clustered based on their overall miRNA expression profiles (fold change).

We evaluated 47 patients with breast cancer. A total of 15 patients were assigned to the cardiac toxicity group. The clinical and histopathological characteristics of all patients are presented in Table 1. No statistically significant differences were observed between two groups (cardiac toxicity vs. non-toxicity) in terms of these characteristics, including age, cancer staging, radiotherapy, medical history, smoking status, body mass index, trastuzumab administration, or total epirubicin dose. Up to 80% of the patients have up to 3 positive lymph nodes and received radiotherapy. Among the 84 miRNAs profiled, 45 showed differential expression between the cardiac toxicity and non-toxicity groups (Figure 1). Of those, 24 were overexpressed in the toxicity group (highest observed fold change between the two groups in hsa-miR-17-5p, hsa-miR-22-3p, hsa-miR-145-5p and hsa-miR-143-3p) while the others are downregulated, all with at least 2-fold difference between groups. Highlighted by downregulation are hsa-miR-155-5p, hsa-miR-124-3p and hsa-miR-133a-3p with fold differences ranging from −4.7 to −3.87. Supplementary Table S1 reports the expression values for all 84 profiled miRNAs and indicates which of them are significantly differentially expressed between the cardiac toxicity and non-toxicity groups. Based on the magnitude of the fold regulation, the five most upregulated miRNAs were hsa-miR-17-5p, hsa-miR-22-3p, hsa-miR-145-5p, hsa-miR-143-3p, and hsa-miR-21-5p, whereas the five most downregulated miRNAs were hsa-miR-155-5p, hsa-miR-124-3p, hsa-miR-133a-3p, hsa-miR-181a-5p, and hsa-miR-210-3p. These ten candidates were selected for further association analyses. The clustering observed in the heatmap provides a biological framework for interpreting these statistical findings (Figure 2).

### 2.2. Association of Top Deregulated miRNAs with Cardiotoxicity

Among the five most downregulated miRNAs described above, higher baseline levels of hsa-miR-155-5p and hsa-miR-124-3p were significantly associated with cardiotoxicity (OR 1.12, 95% CI 1.01–1.23, *p* = 0.03 and OR 1.11, 95% CI 1.01–1.24, *p* = 0.03, respectively) (Table 2). Hsa-miR-181a-5p and hsa-miR-210-3p, which are also part of these five most downregulated miRNAs, showed borderline associations with cardiotoxicity (both OR 1.10, 95% CI 0.99–1.22; *p* = 0.056 and 0.057, respectively).

**Figure 3:**
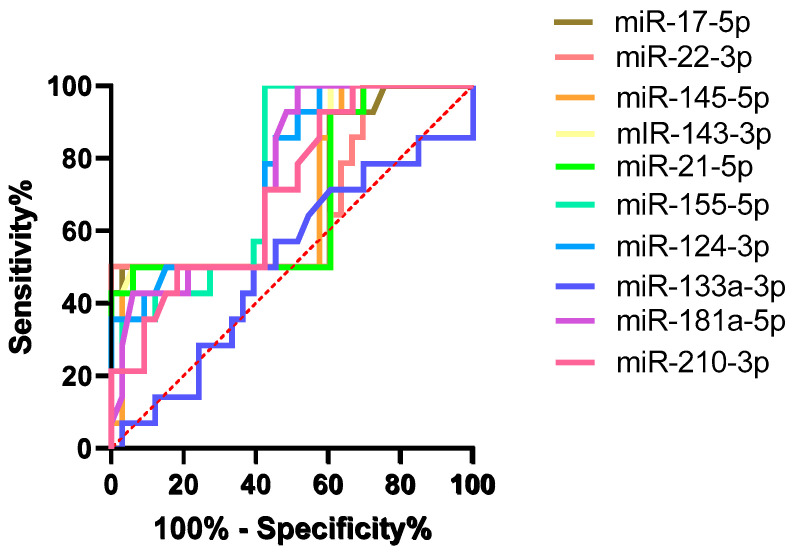
**Receiver operating characteristic (ROC) curves of the top circulating microRNAs for discriminating patients who developed cardiotoxicity from those who did not.** The *y*-axis represents sensitivity (%) and the *x*-axis 100% − specificity (%); the red dashed line indicates the line of no discrimination (AUC = 0.5).

ROC curve analysis demonstrated modest discriminative ability for the upregulated miRNAs (Figure 3), with AUROC values around 0.68–0.69. Among the downregulated miRNAs, hsa-miR-155-5p and hsa-miR-124-3p had the best performance, with AUROC values of 0.76 (*p* = 0.006) and 0.75 (*p* = 0.007), respectively.

### 2.3. Machine Learning Models

The decision tree consisted of three decision nodes, using two key splitting rules to classify samples into non-toxicity or cardiac toxicity (Supplementary Figure S1). The root node split the data based on the expression level of hsa-miR-17-5p (≥27 CT), which was the most influential predictor. Samples with hsa-miR-17-5p ≥ 27 CT were directly classified as non-toxicity with 100% certainty, representing 41% of the total dataset. For samples where hsa-miR-17-5p < 27 CT, a secondary decision was made using hsa-miR-185-5p. If hsa-miR-185-5p ≥ 29 CT, the model classified the sample as cardiac toxicity with 100% certainty, accounting for 51% of the total samples. If hsa-miR-185-5p < 29 CT, the sample was classified as non-toxicity, representing 8% of the dataset.

**Figure 4:**
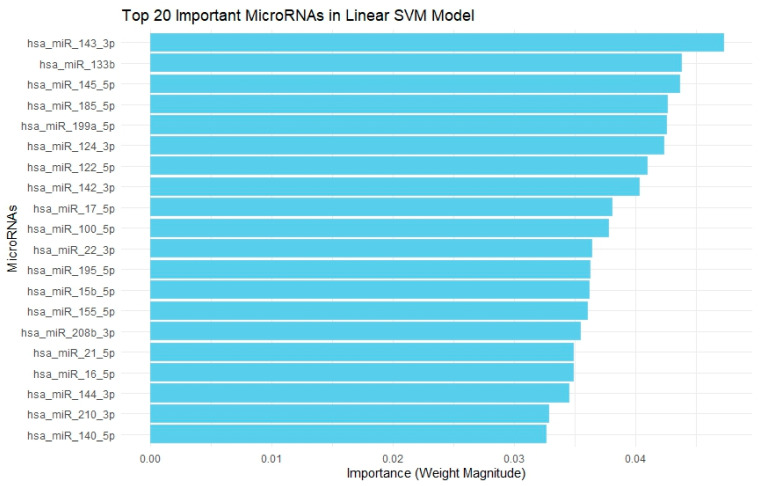
**Top 20 Important MiRNAs concerning cancer therapy related cardiotoxicity in Linear Support Vector Machine Model.** This bar chart displays the top 20 most important miRNAs selected by a Linear Support Vector Machine (SVM) model based on the magnitude of their weight coefficients. The importance reflects the contribution of each miRNA to the classification model, with hsa-miR-143-3p having the highest weight magnitude.

**Figure 5:**
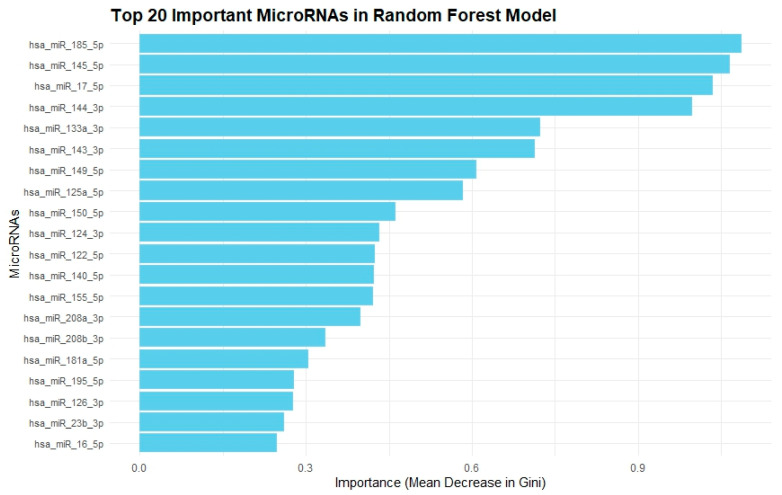
**Top 20 Important MiRNAs concerning cancer therapy related cardiotoxicity in Random Forest Model.** This bar chart presents the top 20 most important miRNAs determined by a Random Forest model, ranked by the mean decrease in Gini impurity. The higher the value, the more important the miRNA is for classification. hsa-miR-185-5p is identified as the most significant feature in this model.

Next, we evaluated 4 other ML models (kNN, SVM, RF, GBM). The metrics of each model are depicted in Table 3, with kNN, SVM, and RF having excellent performance. According to SVM, hsa-miR-143-3p, hsa-miR-133b, hsa-miR-145-5p, hsa-miR-185-5p, and hsa-miR-199a-5p were associated with cardiotoxicity, as shown in Figure 4. According to RF, hsa-miR-185-5p, hsa-miR-145-5p, hsa-miR-17-5p, hsa-miR-144-3p, and hsa-miR-133a-3p were associated with cardiotoxicity, as shown in Figure 5. The KNN, RF, and SVM models were found to have a superior performance in discriminating cardiac toxicity compared to the decision tree (Table 4).

### 2.4. Pathway Analysis

**Figure 6:**
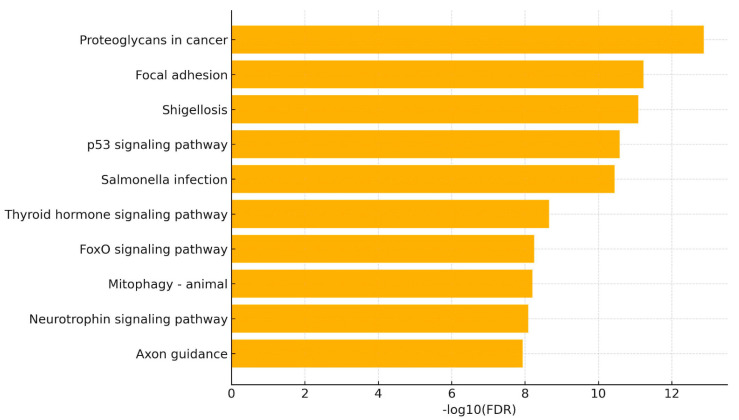
**Pathway enrichment analysis using DIANA-miRPath v4.0.** Analysis was run using TarBase v8.1 as the target database and the KEGG pathway repository for annotation. The “pathways union” mode was selected to identify cumulative pathway enrichments across all miRNAs. Significance was assessed via adjusted *p*-values (false discovery rate (FDR) < 0.05). The top enriched pathways were “Proteoglycans in cancer” (FDR = 1.3 × 10^−13^), “Focal adhesion” (FDR = 5.9 × 10^−12^), “Shigellosis” (FDR = 8.2 × 10^−12^), “p53 signaling pathway” (FDR = 2.7 × 10^−11^), and “Salmonella infection” (FDR = 3.6 × 10^−11^).

The top enriched pathways were “Proteoglycans in cancer” (FDR = 1.3 × 10^−13^), “Focal adhesion” (FDR = 5.9 × 10^−12^), “Shigellosis” (FDR = 8.2 × 10^−12^), “p53 signaling pathway” (FDR = 2.7 × 10^−11^), and “Salmonella infection” (FDR = 3.6 × 10^−11^). Other significantly enriched pathways included “MAPK signaling pathway,” “Pathways in cancer,” and “Apoptosis,” all with FDR values below 1 × 10^−8^. A bar plot of the top 10 pathways, ranked by –log_10_(FDR), is shown in Figure 6. Among the analyzed miRNAs, hsa-miR-17-5p appeared most frequently across enriched pathways, followed by hsa-miR-145-5p and hsa-miR-143-3p. The number of pathways associated with each individual miRNA ranged from 2 to 10, indicating a varying degree of network connectivity and pathway overlap. The number of target genes mapped to each pathway ranged from 8 to 54, depending on the pathway size and miRNA coverage. 

## 3. Discussion

Concerning the unmet need of identifying prognostic biomarkers for antineoplastic regimens-induced cardiotoxicity, for the time being, none of the cardiac biomarkers manage to predict cardiotoxicity with high sensitivity and specificity. MiRNAs are short non-coding single stranded RNAs of 18–25 nucleotides participating in gene expression and regulation of cell differentiation, apoptosis and death [15]. Various miRNAs are implicated in cardiac dysfunction such as heart failure (miR-18a-5p, miR-26b-5p, miR-27a-3p, miR-30e-5p, miR-106a-5p, miR-199a-3p, and miR-652-3p) [16] and acute myocardial infarction (miR-1, miR-21, miR-29a, miR-133a, and miR-208) [17,18].

In our study, we examined the role of 84 miRNAs at baseline as prognostic biomarkers of anthracycline- and trastuzumab-induced cardiotoxicity. The biological rationale for using the Human Cardiovascular Disease Focus V2 panel is grounded in robust evidence connecting specific miRNAs with cardiovascular pathology, tissue specificity, and biomarker value. Some of the included miRNAs (e.g., let-7, miR-1, miR-133, miR-208, miR-126, miR-145, miR-155) are critical regulators of cardiac development, myocardial remodeling, angiogenesis, and vascular function. hsa-miR-17-5p, hsa-miR-22-3p, hsa-miR-145-5p and hsa-miR-143-3p were statistically significantly upregulated (at least a 9-fold), while hsa-miR-155-5p, hsa-miR-124-3p and hsa-miR-133a-3p were downregulated (at least a 14-fold). Several studies investigated the prognostic role of miRNA for anthracycline- and trastuzumab-induced cardiotoxicity with more than 400 miRNAs to be correlated either with down- or upregulation [19,20,21,22,23,24,25,26,27]. The plethora of correlated miRNAs reflects the different mechanisms and pathways that are involved in antineoplastic regimens-induced cardiotoxicity. It is noteworthy that both our study and a recently published pilot study evaluated let-7f-5p, miR-1-3p, and miR-126-3p, observing statistically significant baseline differences in all three miRNAs [28]. However, in our study, baseline miR-126-3p levels were downregulated in patients who developed cardiac toxicity, whereas Pivatto Junior et al. reported high baseline levels of miR-126-3p in this patient group. Furthermore, the significantly reduced baseline levels of let-7f-5p and miR-1-3p observed in our study were not corroborated by the findings of the pilot study.

Additionally, pathway enrichment analysis of the top influential miRNAs identified significant enrichment within signaling networks associated with cardiotoxicity. Enriched signaling networks were focal adhesion, p53 signaling, apoptosis, and MAPK signaling—processes recognized to be involved in cardiac stress, cell killing, and structural remodeling during chemotherapy. The repeated appearance of miR-17-5p, miR-145-5p, and miR-143-3p within these signaling networks implies their core regulatory roles. Infection-related signaling networks, including Shigellosis and Salmonella, were enriched, a result probably indicative of common inflammatory and apoptotic signaling, rather than pathogenesis-specific signaling. These enriched pathways reflect key physiopathological mechanisms of cancer therapy–related cardiotoxicity, including anthracycline-induced DNA damage and p53-mediated apoptosis, oxidative and mitochondrial stress, disruption of cytoskeletal and focal adhesion integrity, and maladaptive MAPK-driven hypertrophic and fibrotic remodeling.

Among the miRNAs studied in relation to anthracycline- and trastuzumab-induced cardiotoxicity, miR-133, miR-1, miR-21, miR-34a, miR-30e and miR-222-3p appear to be highly associated with cardiotoxic effects [20,23,24,25,26,29]. Some of the clinical trials evaluated the timely changed expression of miRNAs either every three months [26] or 15 days [27] or every 3 weeks [20] after the initiation of the treatment. The most common schedule involved assessing miRNAs every 3 months during the first 6 months. However, variations in sampling timepoints may partly explain the wide range of miRNAs found to be associated with cardiotoxicity.

Under the theory of insufficiency of a single biomarker as a prognostic factor, we performed a decision tree, which provided a clear and interpretable decision-making framework, with distinct thresholds for miRNA expression levels determining the classification of cardiac toxicity. This interpretability supports its potential for practical applications in predicting toxicity outcomes based on miRNA profiles. The primary predictor was miR-17-5p, the most significant variable for splitting the data, while the secondary predictor was miR-185-5p, where the expression of miR-17-5p was low. The decision tree highlights the potential of using miRNA profiles for cardiac toxicity classification, providing a simple yet effective tool for distinguishing between toxic and non-toxic outcomes. miR-17-5p is associated with anthracycline-induced cardiotoxicity in pre-clinical and clinical models with controversial results [30,31]. Additionally, while the association of miR-185-5p with antineoplastic regimens-induced cardiotoxicity is unclear, it has been implicated in several cardiac-related processes that could indirectly relate to cardiotoxicity through myocardial fibrosis and angiogenesis [32,33].

Analyzing 4 additional machine learning models, miR-185-5p is identified as the most powerful miRNA related to cardiac toxicity in the RF model, while miR-145-5p in the GBM model and miR-143-3p in the linear SVM model, respectively. Notably, both miR-145-5p and miR-143-3p consistently rank among the most powerful miRNAs across all machine learning models used for cardiac toxicity prediction. The most strongly cardiotoxicity-correlated miRNAs, that emerged in our machine learning models, clustered in the same area in the heatmap. As far as their performance metrics is concerned, all 4 models have better prognostic accuracy in comparison with the decision tree. Although the decision tree provides higher interpretability and might be a straightforward bedside tool, its poor performance reflects a usability-precision trade-off. However, implementation into practice still needs further steps: external validation across independent sets, software tool development to be easily usable, and standardization of miRNA quantitation approaches and miRNA-based machine learning models are only going to be practically valuable if incorporated into a standard, reproducible and cost–benefit format.

Beyond machine learning modeling, we also assessed diagnostic accuracy using logistic regression and ROC analysis, which identified hsa-miR-155-5p and hsa-miR-124-3p as significant predictors. To limit repeated measures error, we focused this analysis on the most strongly differentially expressed miRNAs. The miRNAs identified by ROC, however, differed from those highlighted by machine learning models. This should be interpreted cautiously, as single marker ROC analysis is more sensitive to measurement variability and hierarchical pre-selection, whereas machine learning evaluates multiple miRNAs and is less affected by fluctuations in individual measurements. As a result, ML-based approaches may be more appropriate for interpretation in a miRNA expression profiling study, although further validation is required.

All the above-mentioned miRNAs are associated in the literature with mechanisms that can induce cardiotoxicity. Specifically, the miR-185-5p regulates myocardial fibrosis, angiogenesis and apoptosis, while miR-143-3p inhibits the AKT pathway, all corelated with the cardiotoxicity mechanisms [32,33,34]. Additionally, miR-145-5p and miR-155-5p upregulated in cardiomyocytes after the exposure to doxorubicin [35], while miR-17-5p is associated with anthracycline-induced cardiotoxicity in preclinical and clinical trials with controversial results [30,31,35]. Until now, miR-124 is indirectly connected with chemotherapy-induced cardiotoxicity through its protective role, as it reduces oxidative stress and prevents the cellular apoptosis and autophagy [36,37]. Finally, the levels of miR-22-3p [38] and miR-133a-3p [39] are significantly reduced in cardiomyocytes, which has been exposed to anthracyclines.

Most of these miRNAs are also known to be involved in breast cancer growth. More specifically, miR-185-5p, miR-143-3p, miR-145-5p, miR-124, of miR-22-3p and miR-133a-3p are commonly downregulated in breast cancer as known tumor suppressors, while miR-155-5p and miR-17-5p are considered as oncogenic miRNAs. MiR-185-5p regulated processes like epithelial–mesenchymal transition (EMT) and invasion of breast cancer cells by targeting the receptor for advanced glycation end-products (RAGE) [40], while miR-143-3p inhibits RAS/MEK/ERK and PI3K/AKT/mTOR pathways, which are significant in HER2 downstream signaling [41]. Based on their known roles and expression profiles in our study, miR-185-5p, miR-143-3p, and miR-145-5p emerged as the most consistently identified and biologically relevant candidates across all machine learning models. In contrast, miR-17-5p, miR-124, and miR-133a-3p showed either contradictory behavior or less consistent predictive power, making them less ideal as standalone biomarkers. Overall, the combined presence of miR-185-5p, miR-143-3p, and miR-145-5p underscores their potential relevance and warrants further validation in future studies.

HER2 overexpression that is observed in HER2-positive breast cancer exhibits different miRNA profiles in comparison with HER2-negative tumors and some alterations have been observed according to specific HER2 genotype. For example, miR-33b is significantly downregulated in HER2-positive tumors by targeting MYC and downregulating EZH2 [42], miR-21 activates the RAS/MEK/ERK pathway that is crucial in downstreaming signal of HER2-expression in breast cancer cells [43], while miR-4728 is encoded within an intron of HER2 gene and follows HER2 overexpression by targeting EBP1 and ESR1 [44,45]. Additionally, the aggressive profile of p95HER2 variant includes upregulation of miR-221, miR-222, and miR-503 creating its unique miRNA pattern [46].

While the miRNAs identified in our study have not been strongly linked in the literature to specific HER2 genotypes or overexpression, our dataset did not allow for direct analysis of associations between HER2 expression levels (e.g., IHC staining intensity or mRNA abundance) and miRNA profiles. This represents a key limitation of our work. Future studies incorporating larger patient cohorts and comprehensive HER2 molecular profiling—including IHC scoring, FISH status, and mRNA expression levels—will be crucial to stratify patients effectively. Such stratification may uncover subtype-specific miRNA signatures and improve the precision of cardiotoxicity prediction in HER2-positive breast cancer patients.

Some other limitations of our study are the lack of assessment of miRNAs in different timepoints and the relatively small number of patients with a cardiac event. In addition, the overall sample size was small, which inherently limits the statistical power of our findings and the appropriateness of classic statistical regression analysis. Furthermore, potential experimental confounders—such as inter-individual variability in baseline cardiac function, variability in sample processing time, and batch effects during RNA extraction or qPCR—may have influenced miRNA expression levels. Differences in pre-analytical handling (e.g., plasma isolation time, storage duration) could also contribute to measurement variability. Different methodologies for miRNA quantification and standardization are another barrier for assessing the miRNAs as prognostic tool for cardiotoxicity. Alternative normalization strategies, such as self-normalizing miRNA pairs that do not require a reference miRNA could further improve robustness but would necessitate larger training and validation cohorts and were beyond the scope of this exploratory study. Additionally, based on our study design, we cannot attribute cardiotoxicity to a specific antineoplastic regimen and a direct connection of miRNAs with anthracycline or trastuzumab-induced cardiotoxicity is not possible. Moreover, miRNAs have not been validated in independent cohorts. The absence of external validation further limits the robustness of the results, and the reliance on synthetic data balancing may have introduced potential bias.

Using our findings as the basis to limit the number of examined miRNAs based on specific pathways and to emphasize in AI approaches, large-scale randomized prospective studies including cohorts with anthracyclines or trastuzumab and standardized assessment of miRNAs are needed to confirm the prognostic value of these biomarkers.

## 4. Material and Methods

### 4.1. Patients and Blood Samples

A total of 47 patients with operable breast cancer were evaluated after having surgery and before starting adjuvant chemotherapy with 4 cycles of epirubicin 75 mg/m^2^, given by slow intravenous push during a period of 10 to 15 min and cyclophosphamide 600 mg/m^2^, given by intravenous infusion for 30 to 60 min every 2 weeks and thereafter 12 weekly cycles of paclitaxel 80 mg/m^2^, given by intravenous infusion for 1 h and trastuzumab, at an initial dose of 8 mg/kg as an intravenous infusion over 90 min and subsequent doses of 6 mg/kg as an intravenous infusion over 30–90 min every three weeks. After the completion of chemotherapy, patients continued trastuzumab every 3 weeks for one year in total. Blood samples were collected at baseline and evaluation of cardiac toxicity, through echocardiography and measurement of arterial pressure, was performed every 3 months from treatment initiation until the end of trastuzumab-therapy (15 months), as per common clinical practice and the 2022 ESC Guidelines on cardio-oncology for high-risk patients [47]. The inclusion criteria were (1) Histological confirmation of HER-2 positive breast cancer; (2) indication for adjuvant chemotherapy with anthracyclines and trastuzumab; and (3) left ventricular ejection fraction (LVEF) > 50% at baseline evaluation. The exclusion criteria were (1) Systematic inflammation disease or infection; (2) hepatic or renal failure; (3) coronary disease or LVEF < 50%; (4) serious myocardial disease; and (5) uncontrolled arterial hypertension.

Given the exploratory, pilot nature of this study and the absence of robust prior data to inform expected effect sizes for circulating miRNAs in this specific setting, no formal a priori sample size calculation was performed. Instead, the sample size reflects all consecutive eligible HER2-positive breast cancer patients treated at our institution during the recruitment period who had complete clinical and biospecimen data.

All cases were identified in the Department of Clinical Therapeutics, Alexandra Hospital, National and Kapodistrian University of Athens Medical School between May 2015 and July 2019. Ethical approval for the study was obtained from the Hospital Review Board, and all participants provided written informed consent prior to enrollment, including specific authorization for the collection and use of blood samples and related data for research purposes.

Based on cardiac evaluation, patients were divided into two groups: cardiac toxicity and non-toxicity. Cardiac toxicity was defined as symptomatic decrease in LVEF OR asymptomatic LVEF decrease < 40% OR LVEF decrease ≥ 10% from baseline between 40 and 49% OR LVEF decrease < 10% from baseline between 40 and 49% and Global Longitudinal Strain (GLS) reduction > 15% from baseline or new increase in cardiac biomarkers from baseline [high-sensitivity troponin I (hs-TnI) > 15 pg/mL and NT-proBNP > 125 pg/m] OR LVEF decrease ≥ 50% and GLS reduction > 15% from baseline and/or new increase in cardiac biomarkers from baseline [high-sensitivity troponin I (hs-TnI) > 15 pg/mL and NT-proBNP > 125 pg/m]. Blood samples were collected in serum separator tubes, allowed to clot for 30 min, and afterwards they were centrifuged at 3000 rpm for 10 min at 4 °C. Serum was aliquoted and stored at −80 °C with no repeated freeze–thaw cycles. All analyses were performed using the same reagent batches.

### 4.2. MiRNA Expression

The NucleoSpin miRNA Plasma Kit (Macherey-Nagel, Duren, Germany) was used to isolate circulating RNA from 300 μL of human serum, following the manufacturer’s protocol and reproducing the laboratory’s previously published workflow [48]. RNA concentration was quantified using a Qubit Fluorometer (Thermo Fisher Scientific, Waltham, MA, USA). Typical concentrations were 25–30 ng/μL in a 20 μL elution volume, corresponding to approximately 500–600 ng of total RNA per sample. For reverse transcription, RNA samples were adjusted to 5 ng/μL according to the miRCURY LNA RT Kit recommendations. Each reaction used 4 μL of RNA at 5 ng/μL (20 ng total RNA) in a 20 μL reaction volume, following the protocol for miRCURY LNA Focus Panels (serum/plasma). The expression of 84 miRNAs was then assessed using the miRCURY LNA miRNA PCR Panel, Human Cardiovascular Disease Focus V2 (Cat. no. YAHS-213Y, Qiagen, Hilden, Germany), in combination with the miRCURY LNA SYBR Green Master Mix. Each array contains ten different snoRNA/snRNA as a normalization control for the array data [SNORD44 (hsa), SNORD38B (hsa), SNORD49A (hsa), U6snRNA (v2), UniSP2,3,4,5,6 and cel-miR-39-3p as a spike-in exogenous control], miRNA reverse transcription control (RTC) and positive PCR control (PPC). cel-miR-39-3p was used to monitor RNA extraction and reverse transcription efficiency, ensuring comparability across plasma samples. Endogenous small RNAs (e.g., SNORD44, SNORD49A, U6snRNA) were used for normalization of expression data via the 2^−ΔCt^ method. Samples were grouped into two categories: Cardiac toxicity and non-toxicity. The miRNA relative expression was calculated by the 2^−∆Ct^ method for each miRNA in each sample according to the miRCURY LNA miRNA PCR Data Analysis platform: https://dataanalysis2.qiagen.com/miRCury (accessed on 24 October 2024). Between our groups fold change was calculated with the 2^−∆∆Ct^ method and is represented by fold regulation in a biologically meaningful way, along with the corresponding standard error of mean (SEM) and upper and lower 95% confidence interval (CI). Finally, *p*-values were calculated based on a Student’s *t*-test of the replicate normalized miRNA expression values for each miRNA in the cardiac toxicity and non-toxicity groups and were corrected for false discovery rate (FDR) using the Benjamini–Hochberg method (p.adjust function in R).

### 4.3. Machine Learning Modeling Approach

We assessed five different machine learning models to determine the potential of miRNAs in diagnosing cardiotoxicity in breast cancer patients receiving targeted therapy. The decision tree model was developed to classify cardiac toxicity based on selected miRNA features using the rpart package (version 4.1.24). The methods included data preprocessing, balancing, feature selection, and model construction. To address potential class imbalance, the dataset was balanced using the ROSE (Random Over-Sampling Examples) technique. ROSE generated synthetic samples by combining oversampling of the minority class with undersampling of the majority class, resulting in a balanced dataset. This step aimed to mitigate the bias caused by imbalanced class distributions. The balanced dataset was randomly partitioned into a training set (80%) and a testing set (20%) using stratified sampling to maintain class proportions in both subsets. A 10-fold cross-validation with 10 repetitions was performed on the training dataset to optimize the model and evaluate its generalizability. A Random Forest model was trained on the training dataset to determine the relative importance of the miRNA variables. The model used 500 trees, and the number of randomly selected variables at each split was set to 5. Variable importance was assessed using the Gini index, and the top features were visualized in a variable importance plot. A decision tree model was built using the top three most important miRNA variables identified from the Random Forest analysis.

For the Random Forest (RF), we assessed the importance of each miRNA using the mean decrease in Gini using the randomforest package (version 4.7-1.2). This measures how much including a specific miRNA reduces uncertainty in the decision-making process of the RF; the higher the mean decrease in Gini, the more that miRNA helps to split data effectively and classify outcomes accurately. For the linear Support Vector Machine (SVM) model, a weight magnitude was assigned to each miRNA using the caret package (version 7.0-1); larger magnitudes indicate that a miRNA strongly impacts the decision boundary.

For the k-Nearest Neighbors (kNN) model, data were standardized before training to ensure equal contribution of all features using the class package. The optimal number of neighbors (k) was selected using repeated 10-fold cross-validation. Classification was based on the majority class among the k nearest samples using Euclidean distance. For the Gradient Boosting Machine (GBM) model, an ensemble of decision trees was built sequentially to minimize classification error using the gbm package (version 2.2.2). Key hyperparameters (number of trees, depth, learning rate) were tuned via repeated cross-validation. Feature importance was assessed based on the relative influence of each miRNA across all trees.

### 4.4. MiRNA Pathway Analysis

To explore the biological relevance of the most influential miRNAs associated with cardiotoxicity, we performed pathway enrichment analysis using DIANA-miRPath v4.0: http://www.microrna.gr/miRPathv4 (accessed on 19 July 2025). To identify the most relevant miRNAs across differential expression and ML analyses, we applied an integrative selection strategy. Only miRNAs meeting both statistical significance in differential expression (FDR < 0.05) and high importance ranking (top 5) in any ML model were considered final candidates. Analysis was run using TarBase v8.1 as the target database and the KEGG pathway repository for annotation. The “pathways union” mode was selected to identify cumulative pathway enrichments across all miRNAs. Significance was assessed via adjusted *p*-values (false discovery rate (FDR) < 0.05).

### 4.5. Statistical Analysis

Values are expressed as means ± standard deviation for continuous variables, and as valid percentages for categorical variables. The normality of continuous variables was assessed using the Kolmogorov–Smirnov test and probability–probability (P–P) plots. Between group differences were tested with *t*-test and chi square tests for continuous and categorical variables, respectively. T-tests were conducted to compare miRNAs’ expression between the cardiac toxicity and non-toxicity groups.

To assess the prognostic value of individual miRNAs, we selected the five most upregulated and the five most downregulated miRNAs according to fold regulation between patients with and without cardiotoxicity. For each of these ten miRNAs, a univariate logistic regression model was fitted with cardiotoxicity as the dependent variable and baseline normalized miRNA expression as a continuous predictor. Odds ratios (ORs) with corresponding 95% confidence intervals (CIs) and *p*-values were calculated. In addition, receiver operating characteristic (ROC) curve analysis was performed for each of those miRNAs to evaluate its discriminative ability for cardiotoxicity. The area under the ROC curve (AUROC) and the associated *p*-value were estimated.

For the comparison of different machine learning models, we conducted a one-way analysis of variance (ANOVA) using model accuracy as the dependent variable and model type as the grouping factor. Post hoc pairwise comparisons were performed using Tukey’s Honestly Significant Difference (HSD) test to estimate the mean difference in accuracy between models, along with the corresponding 95% CIs and adjusted *p*-values. All statistical calculations were performed in R studio v. 2024.12.1.

## 5. Conclusions

This study highlights the potential of circulating miRNAs as prognostic biomarkers for therapy-induced cardiotoxicity in HER2-positive breast cancer patients. Among the 84 miRNAs assessed, several showed strong differential expression between patients who developed cardiotoxicity and those who did not. Notably, miR-185-5p, miR-145-5p, and miR-143-3p consistently emerged as top predictors across multiple machine learning models, reinforcing their potential role in risk stratification, while ROC curve analysis revealed modest diagnostic accuracy for hsa-miR-155-5p and hsa-miR-124-3p. The application of machine learning techniques, particularly SVM, kNN, and RF, demonstrated superior predictive performance compared to traditional decision trees, underscoring the value of computational approaches in biomarker-driven cardiotoxicity prediction. These findings support the possible integration of miRNA profiling with AI-based models to enable early identification of high-risk patients and the development of personalized monitoring and intervention strategies. Future large-scale, longitudinal studies with standardized miRNA assessment protocols are needed to validate these results and further refine miRNA-based predictive models for clinical application.

## Figures and Tables

**Table 1:** Clinical and histopathological characteristics of the 47 breast cancer patients.

Characteristics	Cardiac Toxicity (Ν = 15)	Non-Cardiac Toxicity (Ν = 32)	*p*-Value
Age (years)	56.9 ± 12.9	56.3 ± 11.8	0.88
Staging (pTNM)			
I	8 (53.3)	14 (43.8)	0.80
II	5 (33.3)	14 (43.8)	
III	2 (13.3)	4 (12.5)	
Radiotherapy	14 (93.3)	25 (78.1)	0.28
Systolic arterial pressure (mmHg)	119 ± 19	116 ± 18	0.67
Diastolic arterial pressure (mmHg)	78 ± 13	80 ± 11	0.73
Arterial Hypertension	4 (26.6)	11 (34.4)	0.51
Dyslipidemia	6 (39.9)	13 (40.6)	0.97
Diabetes mellitus	1 (6.6)	4 (12.5)	0.67
Non-smokers	10 (66.6)	21 (65.6)	0.91
Past smokers	2 (13.3)	6 (18.8)	
Active Smokers	3 (20)	5 (15.6)	
Body mass index (kg/m^2^)	28.13 ± 4.63	25.94 ± 4.59	0.13
Trastuzumab	11 (73.3)	27 (84.4)	0.34
Total dose epirubicin (mg/m^2^)	515 ± 51	516 ± 49	0.97
Categorical variables are presented as N (%). Continuous variables are presented as mean ± SD			

**Table 2:** Univariate logistic regression and receiver operating characteristic (ROC) curve analyses for the association between baseline expression of the top five upregulated and top five downregulated miRNAs and cardiotoxicity.

miR	OR (95% CI)	*p*-Value	AUROC	*p*-Value
hsa-miR-17-5p	0.92 (0.83–1.03)	0.16	0.69	0.047
hsa-miR-22-3p	0.92 (0.82–1.03)	0.15	0.68	0.06
hsa-miR-145-5p	0.93 (0.84–1.04)	0.19	0.69	0.041
hsa-miR-143-3p	0.93 (0.84–1.04)	0.19	0.69	0.037
hsa-miR-21-5p	0.93 (0.84–1.04)	0.20	0.69	0.045
hsa-miR-155-5p	1.12 (1.01–1.23)	0.03	0.76	0.006
hsa-miR-124-3p	1.11 (1.01–1.24)	0.03	0.75	0.007
hsa-miR-133a-3p	1.02 (0.92–1.12)	0.73	0.51	0.89
hsa-miR-181a-5p	1.10 (0.99–1.22)	0.056	0.75	0.008
hsa-miR-210-3p	1.10 (0.99–1.22)	0.057	0.71	0.025

**Table 3:** Performance metrics of machine learning models.

Model	Accuracy	Precision	Recall	F1	Kappa	Sensitivity	Specificity
k-Nearest Neighbors	0.89	0.88	0.87	0.88	1.00	0.83	0.88
Support Vector Machine	0.92	0.91	0.90	0.91	1.00	0.83	1
Random Forest	0.91	0.90	0.89	0.90	1.00	0.83	0.88
Gradient Boosting	0.93	0.92	0.91	0.92	0.64	1	0.88
Decision Tree	0.75	1.00	0.50	0.67	0.50	0.50	1
Derived from the test dataset (20% of the total sample)							

**Table 4:** Comparison of the assessed machine learning models.

Comparison	Mean Difference in Accuracy (95% CI)	*p*-Value
GBM vs. Decision Tree	0.18 (−0.04, 0.40)	0.13
kNN vs. Decision Tree	0.22 (0.01, 0.44)	0.045
RF vs. Decision Tree	0.24 (0.02, 0.45)	0.03
SVM vs. Decision Tree	0.24 (0.03, 0.46)	0.02
kNN vs. GBM	0.04 (−0.18, 0.26)	0.98
RF vs. GBM	0.06 (−0.16, 0.27)	0.94
SVM vs. GBM	0.06 (−0.15, 0.28)	0.90
RF vs. kNN	0.02 (−0.20, 0.23)	0.99
SVM vs. kNN	0.02 (−0.19, 0.24)	0.99
SVM vs. RF	0.01 (−0.21, 0.23)	0.99

## Data Availability

The original contributions presented in this study are included in the article and Supplementary Material. Further inquiries can be directed to the corresponding author.

## Supplementary Materials

The following supporting information can be downloaded at: https://www.mdpi.com/article/10.3390/ph18121908/s1, Supplementary Figure S1. Decision tree model for cardiac toxicity classification based on miRNA expression levels. The tree uses hsa-miR-17_5p and hsa-miR-185-5p as key predictors, with thresholds determining splits and final classifications into class_0 (Non-Toxicity) or class_1 (Cardiac Toxicity). Each node displays the predicted class, probability of the class, and proportion of samples. Supplementary Table S1. Fold regulations. *p*-values. and FDRs of all 84 miRNAs.
