# Correction: Anastasiou et al. MicroRNA Signatures and Machine Learning Models for Predicting Cardiotoxicity in HER2-Positive Breast Cancer Patients. *Pharmaceuticals* 2025, *18*, 1908

**DOI:** 10.3390/ph19020273

**Published:** 2026-02-06

**Authors:** Maria Anastasiou, Evangelos Oikonomou, Panagiotis Theofilis, Maria Gazouli, George-Angelos Papamikroulis, Athina Goliopoulou, Vasiliki Tsigkou, Vasiliki Skandami, Angeliki Margoni, Kyriaki Cholidou, Amanda Psyrri, Konstantinos Tsioufis, Flora Zagouri, Gerasimos Siasos, Dimitris Tousoulis

**Affiliations:** 1 Section of Medical Oncology, 2nd Propaedeutic Department of Internal Medicine and Clinical Research Institute, Attikon University Hospital, National and Kapodistrian University of Athens Medical School, 11527 Athens, Greece; miriamanastasiou9@gmail.com (M.A.); psyrri237@yahoo.com (A.P.); 2 3rd Department of Cardiology, Sotiria Chest Disease Hospital, National and Kapodistrian University of Athens Medical School, 11527 Athens, Greece; boikono@gmail.com (E.O.); gapapa02@gmail.com (G.-A.P.); agoliopoulou@gmail.com (A.G.); bikytsigkoy@yahoo.gr (V.T.); ger_sias@hotmail.com (G.S.); 3 1st Cardiology Department, “Hippokration” Athens General Hospital, National and Kapodistrian University of Athens Medical School, 11527 Athens, Greece; panos.theofilis@hotmail.com (P.T.); ktsioufis@gmail.com (K.T.); 4 Laboratory of Biology, Department of Basic Medical Sciences, National and Kapodistrian University of Athens Medical School, 11527 Athens, Greece; maria.gazouli@gmail.com; 5 Department of Microbiology, “Hippokration” Athens General Hospital, 11527 Athens, Greece; 6 Department of Biological Chemistry, National and Kapodistrian University of Athens Medical School, 11527 Athens, Greece; angeliki.margoni@gmail.com; 7 1st Respiratory Medicine Department, Sotiria Chest Disease Hospital, National and Kapodistrian University of Athens Medical School, 11527 Athens, Greece; kg.cholidou@yahoo.gr; 8 Department of Clinical Therapeutics, Alexandra Hospital, National and Kapodistrian University of Athens Medical School, 11528 Athens, Greece; florazagouri@yahoo.co.uk

**Text Correction**

In the original publication [1], the ethical approval was missing. Corrections have been made to Section 4.1. Patients and Blood Samples, Paragraph 3, as below:

“Ethical approval for the study was obtained from the Ethics Committee of “Hippokration” Athens General Hospital (Approval Code: 8028; Approval Date: 17 June 2015).”

**Missing Back Matter**

In the original publication [1], the “Institutional Review Board Statement” and “Informed Consent Statement” part were not included. They should be added as below:

**Institutional Review Board Statement:** Ethical approval for the study was obtained from the Ethics Committee of “Hippokration” Athens General Hospital (Approval Code: 8028; Approval Date: 17 June 2015).

**Informed Consent Statement:** Informed consent was obtained from all subjects involved in the study.

The authors state that the scientific conclusions are unaffected. This correction was approved by the Academic Editor. The original publication has also been updated.
