# Supplementary material for: MicroRNA Signatures and Machine Learning Models for Predicting Cardiotoxicity in HER2-Positive Breast Cancer Patients
Source: Pharmaceuticals (Basel). 2025 Dec 18;18(12):1908. doi: 10.3390/ph18121908 (PMC12735735; doi:10.3390/ph18121908)
Supplement: Supplementary file 1 [file pharmaceuticals-18-01908-s001.zip › Supplementary Table.pdf]

Supplementary Table 1. Fold regulations. p-values. and FDRs of all 84 miRNAs.							
**miRNA**	**miRNA family**	**Fold Regulation**	**SEM**	**Upper CI**	**Lower CI**	**P-Value**	**FDR (Adjusted-P-Value)**
hsa-miR-17-5p	mir-17	3.36	0.33	0.62	-0.68	1.3E-11	1.9E-10
hsa-miR-22-3p	mir-22	3.36	0.37	0.11	-1.35	1.4E-11	1.9E-10
hsa-miR-145-5p	mir-145	3.28	0.34	0.24	-1.10	6.9E-13	1.9E-11
hsa-miR-143-3p	mir-143	3.27	0.32	0.64	-0.63	2.3E-13	9.8E-12
hsa-miR-21-5p	mir-21	3.1	0.32	0.06	-1.17	4.2E-12	8.9E-11
hsa-miR-144-3p	mir-144	3.05	0.33	0.26	-1.02	2.4E-11	2.8E-10
hsa-miR-185-5p	mir-185	3.02	0.32	-0.18	-1.42	1.1E-10	1.0E-9
hsa-miR-122-5p	mir-122	2.75	0.32	-0.16	-1.43	3.1E-11	3.2E-10
hsa-miR-146a-5p	mir-146	2.75	0.37	0.56	-0.91	2.4E-8	1.3E-7
hsa-miR-16-5p	mir-15	2.72	0.37	0.77	-0.69	1.3E-6	4.3E-6
hsa-miR-208a-3p	mir-208	2.48	0.37	1.08	-0.39	2.3E-7	8.7E-7
hsa-miR-140-5p	mir-140	2.42	0.37	0.10	-1.34	6.4E-9	4.1E-8
hsa-miR-195-5p	mir-15	2.35	0.38	0.78	-0.73	9.2E-8	3.9E-7
hsa-miR-221-3p	mir-221	2.35	0.36	0.47	-0.93	7.6E-7	2.7E-6
hsa-miR-183-5p	mir-183	2.24	0.38	0.67	-0.81	9.3E-7	3.1E-6
hsa-miR-208b-3p	mir-208	2.24	0.36	0.82	-0.61	2.0E-6	6.3E-6
hsa-miR-100-5p	mir-10	2.22	0.34	0.41	-0.92	8.9E-8	3.9E-7
hsa-miR-149-5p	mir-149	2.22	0.29	0.59	-0.54	8.9E-8	3.9E-7
hsa-miR-30c-5p	mir-30	1.91	0.39	-0.04	-1.58	3.9E-6	1.1E-5
hsa-miR-182-5p	mir-182	1.89	0.37	0.58	-0.87	5.3E-5	1.3E-4
hsa-miR-199a-5p	mir-199	1.86	0.31	0.17	-1.04	1.0E-5	2.7E-5
hsa-miR-342-3p	mir-342	1.49	0.51	0.51	-1.49	6.7E-3	1.3E-2
hsa-miR-142-3p	mir-142	1.39	0.27	0.26	-0.78	4.3E-5	1.1E-4
hsa-miR-365a-3p	mir-365	1.32	0.36	0.43	-0.99	2.1E-3	4.3E-3
hsa-miR-424-5p	mir-322	0.99	0.46	1.25	-0.53	4.8E-2	7.9E-2
hsa-miR-302b-3p	mir-302	0.92	0.61	0.76	-1.65	1.0E-1	1.5E-1
hsa-miR-31-5p	mir-31	0.9	0.45	0.64	-1.14	3.9E-2	6.8E-2
hsa-miR-214-3p	mir-214	0.87	0.49	1.00	-0.94	8.0E-2	1.2E-1
hsa-miR-328-3p	mir-328	0.68	0.48	1.13	-0.77	2.3E-1	3.1E-1
hsa-miR-224-5p	mir-224	0.67	0.46	0.67	-1.13	1.8E-1	2.6E-1
hsa-miR-103a-3p	mir-103	0.58	0.39	0.95	-0.59	1.1E-1	1.7E-1
hsa-miR-29a-3p	mir-29	0.56	0.50	-0.26	-2.24	2.4E-1	3.1E-1
hsa-miR-107	mir-103	0.46	0.34	0.29	-1.04	2.1E-1	2.8E-1
hsa-miR-29b-3p	mir-29	0.46	0.39	0.83	-0.68	2.4E-1	3.1E-1
hsa-miR-26a-5p	mir-26	0.45	0.55	0.78	-1.37	4.1E-1	4.9E-1
hsa-miR-26b-5p	mir-26	0.19	0.76	2.01	-0.99	7.9E-1	8.3E-1
hsa-miR-27b-3p	mir-27	0.16	0.67	-0.27	-2.91	7.9E-1	8.3E-1
hsa-miR-30d-5p	mir-30	0.15	0.27	-0.12	-1.17	5.6E-1	6.5E-1
hsa-miR-25-3p	mir-25	0.03	0.41	0.23	-1.38	9.2E-1	9.5E-1

Supplementary Table 1. Fold regulations. p-values. and FDRs of all 84 miRNAs.							
**miRNA**	**miRNA family**	**Fold Regulation**	**SEM**	**Upper CI**	**Lower CI**	**P-Value**	**FDR (Adjusted-P-Value)**
hsa-let-7a-5p	let-7	-0.04	0.29	0.61	-0.53	9.5E-1	9.6E-1
hsa-miR-451a	mir-451	-0.04	0.40	0.30	-1.26	9.6E-1	9.6E-1
hsa-miR-99a-5p	mir-10	-0.12	0.34	-0.22	-1.53	7.9E-1	8.3E-1
hsa-miR-130a-3p	mir-130	-0.15	0.35	0.90	-0.48	7.2E-1	7.8E-1
hsa-let-7b-5p	let-7	-0.18	0.41	0.42	-1.17	6.9E-1	7.6E-1
hsa-miR-423-3p	mir-423	-0.18	0.39	0.96	-0.57	6.8E-1	7.6E-1
hsa-miR-29c-3p	mir-29	-0.18	0.57	0.93	-1.29	8.0E-1	8.3E-1
hsa-miR-93-5p	mir-17	-0.28	0.37	0.95	-0.51	5.4E-1	6.4E-1
hsa-miR-92a-3p	mir-25	-0.28	0.54	0.64	-1.50	6.8E-1	7.6E-1
hsa-miR-23a-3p	mir-23	-0.38	0.65	0.94	-1.60	6.1E-1	7.0E-1
hsa-miR-7-5p	let-7	-0.4	0.27	0.37	-0.68	2.0E-1	2.8E-1
hsa-miR-486-5p	mir-486	-0.43	0.38	0.95	-0.53	3.0E-1	3.8E-1
hsa-miR-98-5p	let-7	-0.52	0.55	0.71	-1.47	4.3E-1	5.1E-1
hsa-let-7c-5p	let-7	-0.63	0.28	0.42	-0.66	3.7E-2	6.6E-2
hsa-miR-494-3p	mir-154	-0.65	0.64	1.19	-1.32	3.8E-1	4.6E-1
hsa-miR-30e-5p	mir-30	-0.68	0.37	0.95	-0.49	7.3E-2	1.1E-1
hsa-miR-499a-5p	mir-499	-0.69	0.51	0.77	-1.22	2.4E-1	3.1E-1
hsa-miR-206	mir-1	-0.74	0.51	1.07	-0.92	1.3E-1	1.8E-1
hsa-miR-24-3p	mir-24	-0.9	0.43	1.36	-0.34	7.7E-2	1.2E-1
hsa-miR-223-3p	mir-223	-0.91	0.44	0.55	-1.19	5.3E-2	8.6E-2
hsa-miR-10b-5p	mir-10	-0.92	0.35	0.19	-1.18	1.8E-2	3.3E-2
hsa-miR-320a-3p	mir-320	-0.98	0.48	0.56	-1.31	6.9E-2	1.1E-1
hsa-miR-378a-3p	mir-378	-1.02	0.31	0.14	-1.06	3.5E-3	6.8E-3
hsa-miR-30a-5p	mir-30	-1.03	0.43	0.55	-1.14	4.2E-2	7.1E-2
hsa-miR-15b-5p	mir-15	-1.5	0.33	0.30	-1.00	1.9E-4	4.4E-4
hsa-miR-27a-3p	mir-27	-1.55	0.45	0.62	-1.13	2.6E-3	5.2E-3
hsa-miR-125b-5p	mir-10	-1.59	0.35	0.57	-0.81	5.1E-4	1.1E-3
hsa-miR-18b-5p	mir-17	-1.7	0.72	2.50	-0.30	4.4E-2	7.3E-2
hsa-miR-302a-3p	mir-302	-1.72	0.62	1.02	-1.42	1.5E-2	2.9E-2
hsa-let-7e-5p	let-7	-2.05	0.53	1.30	-0.80	1.3E-3	2.7E-3
hsa-let-7d-5p	let-7	-2.17	0.37	0.34	-1.12	4.8E-6	1.3E-5
hsa-miR-1-3p	mir-1	-2.24	0.48	1.09	-0.79	1.4E-4	3.3E-4
hsa-miR-222-3p	mir-221	-2.36	0.55	1.38	-0.77	2.2E-4	4.9E-4
hsa-miR-126-3p	mir-126	-2.86	0.35	0.27	-1.11	2.0E-8	1.1E-7
hsa-miR-150-5p	mir-150	-2.87	0.34	0.52	-0.82	1.6E-10	1.3E-9
hsa-miR-125a-5p	mir-10	-2.87	0.43	1.67	0.00	2.9E-5	7.5E-5
hsa-let-7f-5p	let-7	-3.07	0.39	0.79	-0.73	1.8E-8	1.1E-7
hsa-miR-133b	mir-133	-3.16	0.39	0.84	-0.71	2.7E-8	1.3E-7
hsa-miR-181b-5p	mir-181	-3.32	0.42	1.14	-0.52	2.5E-6	7.5E-6

Supplementary Table 1. Fold regulations. p-values. and FDRs of all 84 miRNAs.							
**miRNA**	**miRNA family**	**Fold Regulation**	**SEM**	**Upper CI**	**Lower CI**	**P-Value**	**FDR (Adjusted-P-Value)**
hsa-miR-23b-3p	mir-23	-3.35	0.47	1.19	-0.67	3.4E-6	9.7E-6
hsa-miR-210-3p	mir-210	-3.43	0.45	0.90	-0.86	7.8E-7	2.7E-6
hsa-miR-181a-5p	mir-181	-3.55	0.38	0.45	-1.06	5.3E-10	3.7E-9
hsa-miR-133a-3p	mir-133	-3.87	0.35	-0.13	-1.49	5.7E-15	4.8E-13
hsa-miR-124-3p	mir-124	-4.04	0.34	0.41	-0.94	3.3E-10	2.5E-9
hsa-miR-155-5p	mir-155	-4.7	0.53	1.72	-0.37	1.3E-7	5.1E-7
SEM: standard error of mean, CI: confidence interval, FDR: False discovery rate							
