# Supplementary figures and images for: MicroRNA Signatures and Machine Learning Models for Predicting Cardiotoxicity in HER2-Positive Breast Cancer Patients
Source: Pharmaceuticals (Basel). 2025 Dec 18;18(12):1908. doi: 10.3390/ph18121908 (PMC12735735; doi:10.3390/ph18121908)

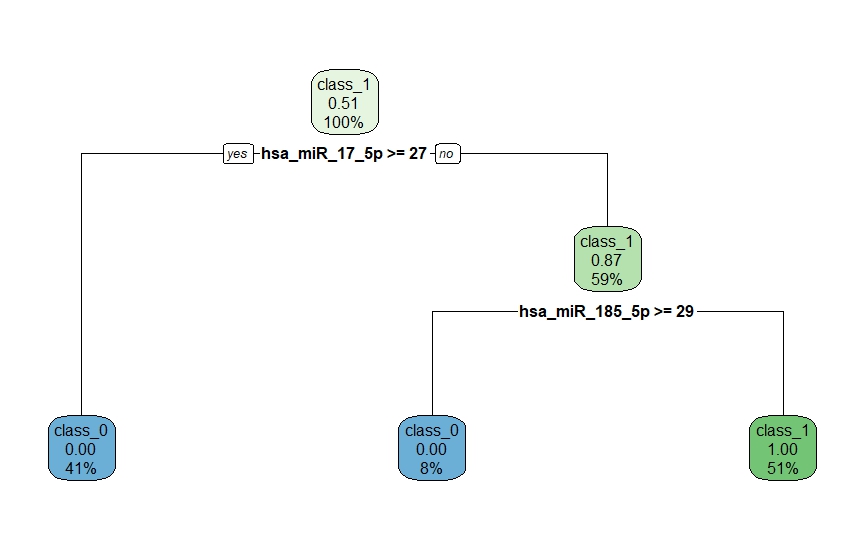

Supplement: Supplementary file 1 [file pharmaceuticals-18-01908-s001.zip › Supplementary figure 1.jpeg]
